# DOU-Pose: Robust Camera-Based Visual Localization for Autonomous Vehicles in Repetitive and Low-Texture Intelligent Transportation Environments

**DOI:** 10.3390/s26165070

**Published:** 2026-08-10

**Authors:** Xin’an Qiu, Liwen Wang, Zezheng Dong, Xiao Xiao, Zhihao Liu, Lin Zhu, Jingyin Wang, Hao Xu

**Affiliations:** 1 Lanzhou Institute of Physics, Lanzhou 730000, China; qxa_608@163.com; 2 School of Telecommunications Engineering, Xidian University, Xi’an 710071, China; liwenwang@stu.xidian.edu.cn (L.W.); zhu_lin@stu.xidian.edu.cn (L.Z.); jingyinwang@stu.xidian.edu.cn (J.W.); xxxh@stu.xidian.edu.cn (H.X.); 3 Shanghai Institute of Satellite Engineering, Shanghai 201109, China; 24011211194@stu.xidian.edu.cn; 4 School of Missile Engineering, Rocket Force University of Engineering, Xi’an 710025, China; liuzh_epgc@163.com

**Keywords:** visual localization, autonomous driving, pose estimation, depthwise over-parameterized convolution, differentiable RANSAC, RGB-D sensing

## Abstract

Accurate and robust vehicle localization is essential for autonomous driving. However, existing visual pose estimation methods often struggle in scenarios dominated by repetitive structures or sparse textures. These conditions lead to ambiguous predictions of 3D scene coordinates and a high proportion of structured outliers—erroneous predictions forming coherent clusters that deceive standard estimators. To address these limitations, this paper proposes DOU-Pose (Depthwise Over-parameterized U-shaped Pose estimation), a visual pose estimation framework built upon the Differentiable SAmple Consensus (DSAC)* pipeline. The core idea is to enhance the discriminative capability of scene coordinate regression through improved feature extraction. Specifically, we replace standard convolutional layers with Depthwise Over-parameterized Convolution (DO-Conv), which introduces auxiliary learnable depthwise kernels during training to enrich the representational capacity of the network, while allowing their fusion into a single kernel for inference. Furthermore, a U-shaped regression network with transposed convolutions is designed to preserve spatial details and strengthen fine-grained geometric reasoning. The entire pipeline is trained end-to-end by coupling dense scene coordinate prediction with a differentiable robust estimator. Extensive experiments demonstrate that DOU-Pose achieves competitive performance on public benchmarks and clear robustness improvements on the self-collected Campus-AV dataset, especially in repetitive and low-texture outdoor driving scenarios.

## 1. Introduction

Precise and robust vehicle localization is a fundamental prerequisite for the safe operation of autonomous mobile robots and Intelligent Transportation Systems (ITSs). While multi-sensor fusion systems (GNSS/LiDAR/IMU) are commonly employed in practice, their reliability can be significantly degraded in complex urban environments, such as urban canyons and tunnels, where satellite signals are blocked or severely affected by multipath interference. In such cases, visual pose estimation provides an important complementary solution by estimating the six-degrees-of-freedom (6-DoF) camera pose relative to a pre-mapped scene [1].

In recent years, learning-based visual localization has achieved substantial progress. In particular, scene coordinate regression (SCR) methods, such as DSAC++ [2] and DSAC* [3], have demonstrated strong localization accuracy by training a neural network to predict the 3D world coordinate associated with each 2D pixel in the query image. These dense 2D-3D correspondences are then used to estimate the camera pose through the Perspective-n-Point (PnP) [4,5] algorithm within a RANSAC framework. By combining learned correspondence prediction with geometric optimization, SCR methods offer an effective balance between accuracy and robustness.

However, despite their strong performance in indoor benchmarks and relatively controlled scenes, directly applying SCR methods to real-world autonomous driving remains challenging. Compared with indoor environments, driving scenes exhibit stronger visual ambiguity, larger appearance variation, and more severe structural repetition. As a result, the scene coordinate regression network is more likely to generate ambiguous predictions and structured outliers, which can substantially degrade downstream pose estimation.

One major challenge comes from repetitive structures that frequently appear in transportation environments. Examples include highway guardrails, building facades with similar windows, fences, road boundaries, and lane markings. These patterns often exhibit strong local similarity but correspond to different global 3D locations. For a standard CNN-based regression network with limited local receptive fields, distinguishing such regions based only on local appearance is difficult. A local image patch from one segment of a guardrail, for instance, may look almost identical to another patch several meters away. In this case, the network may fail to assign a unique and correct scene coordinate to the corresponding pixels, and instead produce ambiguous predictions or regress toward incorrect intermediate positions. Such errors are particularly problematic because they are not isolated random outliers; rather, they often form coherent geometric structures that can still appear plausible to a robust estimator, thereby increasing the risk of pose failure.

Another important challenge arises from sparse-texture or textureless regions, which are also common in outdoor driving scenes. Large portions of the image may be occupied by uniform asphalt, clear sky, overexposed surfaces, or weakly textured building walls under dynamic illumination. In these regions, discriminative image cues are limited, making it difficult for the network to infer stable correspondences from appearance alone. As a result, the predicted scene coordinates can become noisy, unstable, or highly sensitive to small image perturbations. When such unreliable predictions occupy a sufficiently large portion of the image, the number of valid correspondences available for pose estimation is reduced, and the overall robustness of the PnP-RANSAC pipeline deteriorates noticeably. Therefore, improving the quality of scene coordinate prediction in these visually ambiguous regions is crucial for reliable localization in autonomous driving.

To address these limitations, this paper proposes a robust visual pose estimation framework, termed **DOU-Pose** (Depthwise Over-parameterized U-shaped Pose estimation). Rather than relying solely on downstream robust estimation to reject erroneous correspondences, we aim to improve the quality of scene coordinate prediction itself by enhancing both feature discrimination and spatial-detail recovery. Specifically, we introduce Depthwise Over-parameterized Convolution (DO-Conv) [6] into the regression backbone to strengthen feature representation under perceptual aliasing and weak textures, while folding the auxiliary kernel parameterization for inference. In addition, we redesign the regression network as a U-shaped architecture with transposed convolutions and skip connections, allowing deep contextual features to be combined with shallow high-resolution cues for more spatially precise scene coordinate prediction.

By embedding these improvements into a differentiable RANSAC framework, the proposed method enables end-to-end training from dense scene coordinate regression to final camera pose estimation. This integration combines enhanced scene-coordinate prediction with the geometry-based pose-estimation structure of SCR localization for visually ambiguous outdoor scenes.

The main contributions of this paper are summarized as follows:

- We develop **DOU-Pose** for robust visual localization in repetitive and low-texture scenes under the scene coordinate regression and differentiable robust pose-estimation paradigm of DSAC*.
- We redesign the regression network with a DO-Conv encoder and a U-shaped decoder with transposed convolutions and skip connections to improve feature discrimination and spatial-detail recovery. We additionally use grayscale input preprocessing and support RGB-D pose estimation through Kabsch alignment of 3D–3D correspondences.
- We construct an extensive real-world vehicular dataset using a connected autonomous vehicle platform equipped with ZED 2i cameras. Comprising 50,000 synchronized frames collected from a university campus environment, this dataset covers diverse and challenging scenarios. Across three training seeds, DOU-Pose reduces the mean translation and rotation errors relative to DSAC* by 11.2% and 4.4%, respectively, on the held-out Campus-AV test loop.

## 2. Related Work

Within intelligent transportation systems, localization complements environmental perception. Viadero-Monasterio et al. [7] combine frequency-modulated continuous-wave radar distance and reflected power to distinguish pedestrians, vehicles, and irrelevant objects, reporting 91.1% identification accuracy for driving assistance.

Visual pose estimation spans geometric, learning-based, and hybrid methods. Geometric pipelines establish 2D–3D correspondences with SIFT [8], SURF [9], or ORB [10], recover pose through PnP variants [11,12,13], and reject outliers with RANSAC [14]. SfM reconstructs scene maps from multi-view geometry [15,16,17]. For large-scale SfM models, cascaded parallel filtering reduces memory demands during image localization and suppresses erroneous matches [18]. Self-supervised fine-grained segmentation improves long-term visual localization across seasonal and appearance changes [19]. Image retrieval [20,21,22,23,24] and hashing [25,26,27] provide scalable coarse localization, although appearance matching remains vulnerable to repetitive, low-texture, and illumination-varying scenes.

Absolute pose regression (APR) predicts pose from an RGB image. PoseNet [1] adopts the GoogLeNet/Inception architecture as its image feature backbone [28]. Subsequent APR methods explored uncertainty [29], LSTM and video context [30,31], hourglass networks [32], and lightweight decision forests [33]. Semantic object relations [34] and relative geometric constraints [35] further introduce scene structure. MaRePo instead conditions a scene-agnostic pose regressor on a scene-specific map representation, anchoring its predictions to the mapped environment [36].

Hybrid approaches combine learned correspondences with geometric pose estimation. Scene coordinate regression (SCR) predicts a 3D coordinate for each pixel and then applies PnP. Early random-forest regressors [37,38,39] were followed by differentiable DSAC [40], DSAC++ [2], hierarchical prediction [41], and DSAC* [3]. ACE separates a scene-agnostic encoder from a scene-specific head for rapid training [42], while VS-Net introduces segmentation and voting for outdoor robustness [43]. Probabilistic formulations have also modeled pose uncertainty through rotation distributions such as the matrix Fisher distribution [44].

Recent SCR methods emphasize scene representation, spatial context, and supervision quality. NeuMap combines compact scene codes with a scene-agnostic Transformer decoder [45]; GLACE propagates co-visibility through global–local encoding and feature diffusion [46]; and reprojection-error prompts guide region selection during training [47]. R-SCoRe integrates co-visibility encoding, augmentation, and depth-adjusted reprojection loss for large-scale scenes with illumination and image-level ambiguities [48]. These directions complement DOU-Pose, which strengthens SCR feature extraction and multi-scale fusion for repetitive and low-texture regions.

Repetitive structures and sparse textures can still produce ambiguous scene-coordinate predictions and reduce pose accuracy. DOU-Pose therefore targets the SCR backbone to improve feature discrimination and spatial-detail recovery under these conditions.

## 3. Preliminaries

Before presenting our proposed DOU-Pose framework, we formally define the visual localization problem and briefly review the mathematical foundations of scene coordinate regression and Depthwise Over-parameterized Convolution, which serve as the building blocks of our method.

### 3.1. Problem Formulation

The goal of visual pose estimation is to determine the absolute 6-DoF pose of a camera, h, relative to a known world coordinate system, given a query image *I*. The pose h consists of a 3D rotation matrix R∈SO(3) and a 3D translation vector $t\in R^{3}$, transforming a point from the camera coordinate system to the world coordinate system. For a pixel *i* in the image domain, let $p_{i}\in R^{2}$ denote its 2D position and $e_{i}\in R^{3}$ denote its corresponding 3D coordinate in the camera space. The relationship between the scene coordinate $y_{i}\in R^{3}$ (point in world space) and the camera coordinate is defined as:

(1) $$y_{i}=h\cdot e_{i}=Re_{i}+t$$

Ideally, if we can predict the scene coordinates $Y=\{y_{i}\}$ for all pixels, the camera pose h can be recovered by solving the Perspective-n-Point (PnP) problem effectively.

### 3.2. Scene Coordinate Regression via DSAC*

Our approach builds upon the DSAC* framework [3], which treats localization as a two-stage learning process. First, a Fully Convolutional Network (FCN), denoted as f(·;w), maps the input image *I* to dense scene coordinates

(2) Y=f(I;w)

where w represents the learnable network parameters. Second, a differentiable RANSAC estimator selects the best pose hypothesis by maximizing a soft inlier count. Unlike traditional RANSAC, which uses non-differentiable *argmax* operations, DSAC* employs probabilistic selection to allow gradients to backpropagate from the pose loss to the scene coordinate regression network, enabling end-to-end training.

### 3.3. Depthwise Over-Parameterized Convolution (DO-Conv)

To enhance feature extraction in ambiguous scenes, we replace standard convolutional layers with Depthwise Over-parameterized Convolution (DO-Conv) [6].

A standard convolutional layer applies a learnable kernel $W\in R^{C_{out}\times C_{in}\times K\times K}$ to an input feature map P, producing an output O. DO-Conv over-parameterizes this process during the training phase by introducing an auxiliary depthwise convolution kernel $D\in R^{\left(C_{in}\times K\times K\right)\times D_{mul}}$, where $D_{mul}$ is the depth multiplier. For all DO-Conv layers in DOU-Pose, we set $D_{mul}=9$, enable learnable biases, and omit normalization layers.

Formally, the DO-Conv operation is mathematically equivalent to convolving the input with a “folded” kernel. Specifically, the depthwise kernel D is first convolved with the standard kernel W to form an equivalent kernel $W^{\prime }$. Let $D^{T}$ denote the transpose of D reshaping the depthwise interaction. The folded kernel $W^{\prime }$ used for inference is derived as

(3) $$W^{\prime }=D^{T}⊛W$$

where ⊛ denotes the depthwise convolution operation applied to the kernel space.

Consequently, the output feature map is computed as

(4) $$O=W^{\prime }*P$$

where ∗ denotes the standard convolution operator. This formulation, Equations (3) and (4), ensures that while the training phase benefits from the depthwise over-parameterization introduced by D, the inference phase relies solely on the folded kernel $W^{\prime }$ and executes it as a standard convolution.

## 4. Methodology

In this section, we present the details of our proposed DOU-Pose framework. We first articulate the motivation behind our architectural choices, then outline the system pipeline and specific network components. To place our design in context, we follow the scene coordinate regression (SCR) paradigm for 6-DoF visual localization: given a query image, the network predicts dense 2D-3D correspondences (scene coordinates), which are subsequently converted into the camera pose h=(R,t) via a differentiable RANSAC/PnP procedure as in DSAC* [3]. In RGB mode, DOU-Pose constructs 2D-3D correspondences and performs PnP pose-hypothesis generation, soft-inlier scoring, differentiable hypothesis selection, and pose refinement following DSAC*. The SCR network couples a DO-Conv encoder [6] with a U-shaped decoder to improve scene-coordinate prediction in repetitive and low-texture regions. The framework also uses grayscale input preprocessing and supports RGB-D estimation by applying Kabsch alignment to 3D–3D correspondences.

### 4.1. System Overview

**Figure 1:**
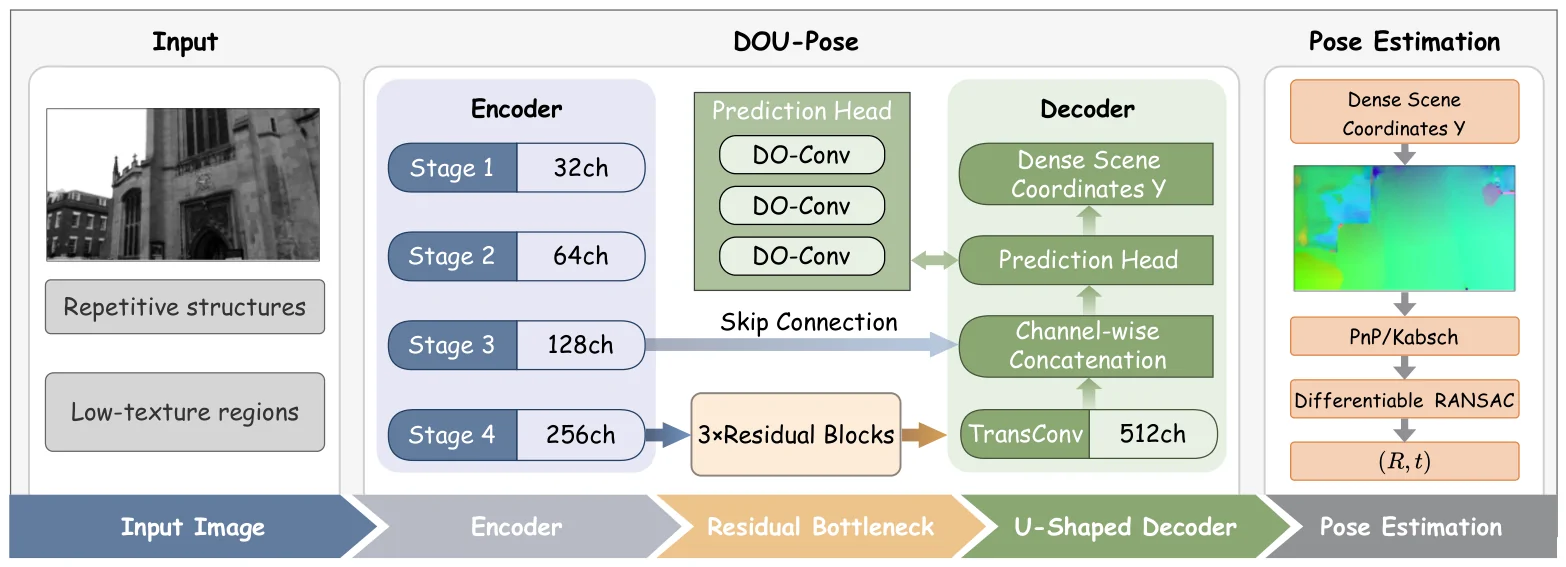
The architecture of the proposed DOU-Pose framework. The network adopts a U-shaped structure, replacing standard convolutions with **DO-Conv** layers in the encoder to enhance feature extraction. **Transposed convolutions** are employed in the decoder to upsample feature maps and recover spatial details. The network predicts dense scene coordinates, which are subsequently used to solve the camera pose via a differentiable RANSAC loop.

The overall pipeline of our method is illustrated in Figure 1. The system takes a single image *I* as input. To enhance robustness against non-linear illumination changes frequently encountered in dynamic driving scenarios—such as tunnel entries/exits and alternating shadows from roadside vegetation—we convert RGB images to grayscale before feeding them into the network. The core component is a fully convolutional network that predicts the dense 3D scene coordinates Y on a regularly subsampled grid at one-quarter of the input resolution in each spatial dimension. These predictions are then passed to a differentiable RANSAC estimator, which samples minimal subsets of 2D-3D correspondences to generate pose hypotheses. The final camera pose h is obtained by refining the best hypothesis selected via soft inlier counting.

Given an input image *I*, DOU-Pose predicts dense scene coordinates $Y=\{y_{i}\}$ with the SCR network f(·;w). Algorithm 1 then estimates the pose using DSAC*: we generate and score multiple hypotheses, select the best one, and refine it to obtain $h_{est}$. We use PnP with reprojection residual $r_{RGB}$ for RGB inputs and Kabsch with 3D residual $r_{RGB-D}$ for RGB-D inputs.
**Algorithm 1** DOU-Pose inference (forward pass)**Require:** Image *I*, intrinsics *K*, (optional) depth map; SCR net f(·;w); hypotheses *N*; soft inlier threshold.
**Ensure:** Pose $h_{est}=\left(R_{est},t_{est}\right)$.
  1:$I\leftarrow G_{\mathrm{RAY}}(I)$; Y←f(I;w)                                                                                                                      (Equation (2))  2:Build correspondences C: RGB: $C=\{\left(p_{i},y_{i}\right)\}$; RGB-D: $e_{i}\leftarrow B_{\mathrm{ACKPROJECT}}\left(p_{i},\mathrm{depth},K\right)$, $C=\{\left(e_{i},y_{i}\right)\}$.  3:**for** j=1 **to** *N* **do**  4:   Sample minimal set $S_{j}\subset C$ with size m=4 (RGB) or m=3 (RGB-D).  5:   Estimate pose $h_{j}\leftarrow \left\{\begin{array}{cc} P_{N}P(S_{j},K) & \mathrm{RGB}\\ K_{\mathrm{ABSCH}}(S_{j}) & \mathrm{RGB}-D \end{array}\right.$  6:   Compute residuals $\left\{r_{i}\right\}$ over C using $r_{RGB}$ or $r_{RGB-D}$.  7:   Score $s\left(h_{j}\right)\leftarrow \mathrm{DSAC}*S_{\mathrm{OFT}}I_{\mathrm{NLIER}}\left(\left\{r_{i}\right\}\right)$.  8:**end for**  9:$h_{best}\leftarrow \arg\max_{h_{j}}s\left(h_{j}\right)$                                                                                              (hard selection at inference)10:Refine $h_{best}$: minimize $\sum_{i}r_{RGB}\left(y_{i},h\right)$ (RGB, LM) or $\sum_{i}r_{RGB-D}\left(y_{i},h\right)$ (RGB-D).11:**return** $h_{est}\leftarrow h_{best}$.


### 4.2. DOU-Pose Network Architecture

The core of our pipeline is the scene coordinate regression (SCR) network. While previous approaches often rely on modified ResNet backbones, we argue that such architectures, originally designed for classification, may lose critical spatial details due to aggressive downsampling. Inspired by the U-Net architecture widely used in biomedical image segmentation [49], we design a U-shaped fully convolutional network that effectively combines deep semantic features with shallow high-resolution details. The detailed configuration of our network is illustrated in Figure 1, comprising three main components: a DO-Conv encoder, a residual bottleneck, and a transposed convolution decoder.

#### 4.2.1. Encoder with DO-Conv

The encoder serves as the feature extraction backbone, responsible for capturing local geometric patterns from the input image. Unlike standard architectures that use conventional convolutional layers, our encoder is constructed using four stages of Depthwise Over-parameterized Convolution (DO-Conv) blocks with output channels of 32, 64, 128, and 256, respectively. Specifically, each encoder block employs 3×3 filters. To introduce non-linearity and mitigate the vanishing gradient problem, we utilize ReLU (Rectified Linear Unit) after each hidden convolution. The ReLU activation also encourages sparsity in the learned representations, which helps in suppressing noise from the background. Downsampling is performed after the first three stages using 3×3 max-pooling layers with a stride of 2 and padding of 1. This progressively reduces the spatial resolution to capture higher-level semantic context while expanding the receptive field. By replacing standard convolutions with DO-Conv, we increase the number of learnable parameters during training through the depthwise multiplier, while kernel folding removes the auxiliary DO-Conv parameterization from the inference graph.

#### 4.2.2. Bottleneck with Residual Blocks

Following the encoder, the network enters a bottleneck phase. Here, we employ three consecutive Residual Blocks (ResBlocks) with input–output channel configurations of 256-to-512, 512-to-512, and 512-to-512. Each ResBlock consists of two convolutional layers and a skip connection between its input and output. These residual connections are crucial for two reasons: first, they facilitate the flow of gradients during backpropagation, allowing for the training of deeper networks; second, they enable the fusion of features from different depths, ensuring that the network retains both the high-level semantic information extracted by the encoder and the structural integrity required for precise coordinate regression.

#### 4.2.3. Decoder and Transposed Convolution

The decoder contains one 512-to-512 transposed convolution and fuses the corresponding skip features by channel-wise concatenation. We utilize this transposed convolution with a 4×4 kernel size and a stride of 2 and padding of 1 to upsample the feature maps by a factor of 2. Unlike simple bilinear upsampling, the transposed convolution contains learnable parameters, allowing the network to learn how to optimally reconstruct spatial details from the bottleneck features. This upsampling process effectively creates a U-shaped structure, where the “What” (semantic content) from the bottleneck is merged with the “Where” (spatial location) reconstructed by the decoder.

#### 4.2.4. Prediction Head and Design Choices

The final stage of the network is the prediction head. We employ three DO-Conv layers with configurations of 512-to-512 channels using a 1-by-1 kernel, 512-to-512 channels using a 1-by-1 kernel, and 512-to-3 channels using a 3-by-3 kernel. The first two layers use ReLU, and the final layer is linear. The 3-channel output corresponds to the (x,y,z) scene coordinates in the world frame and has a resolution of 1/4 of the original input image.

It is worth noting two specific design choices we made based on empirical observations:

- **Grayscale Input**: We convert each RGB image to a single-channel grayscale image before feeding it into the network. This preprocessing emphasizes intensity-defined structural cues and provides a consistent input representation under changing illumination.
- **Fully Convolutional Design**: We eschew traditional fully connected layers in favor of a fully convolutional architecture. This not only reduces the memory footprint but also allows the network to handle input images of arbitrary sizes during inference.

### 4.3. Differentiable Pose Optimization

Given the predicted scene coordinates Y, the final camera pose h is estimated via a differentiable RANSAC pipeline. As described in the DSAC* framework, this process involves three main steps:

- **Hypothesis Sampling**: We randomly sample *M* minimal subsets of correspondences from the dense prediction Y. For each subset, a pose hypothesis $h_{j}$ is computed using a geometric solver g(·).
- **Hypothesis Selection**: To make the selection differentiable, we score each hypothesis $h_{j}$ using a soft inlier count. The probability of selecting a hypothesis is calculated via a Softmax distribution over these scores.
- **Refinement**: The selected best hypothesis $h_{best}$ serves as an initialization. We then refine it using all valid inliers to obtain the final pose $h_{final}$.

We employ tailored solvers g(·) and refinement strategies depending on the input modality:

#### 4.3.1. RGB-Based Estimation (PnP)

When only RGB images are available, the 2D-3D correspondences $C_{RGB}=\left\{\left(p_{i},y_{i}\right)\right\}$ are used. We utilize the **Perspective-n-Point (PnP)** algorithm as the solver g(·) [4,5]. In the sampling step, we draw minimal subsets of 4 correspondences. In the refinement step, the selected hypothesis is optimized by minimizing the reprojection error using the Levenberg–Marquardt (LM) algorithm. The residual function is defined as

(5) $$r_{RGB}\left(y_{i},h\right)={∥p_{i}-Kh^{-1}y_{i}∥}_{2}$$

where *K* denotes the camera intrinsic matrix and $p_{i}$ is the 2D pixel observation.

#### 4.3.2. RGB-D Based Estimation (Kabsch)

When depth information is available (RGB-D mode), we can back-project the 2D pixels to 3D camera coordinates $e_{i}$ using the depth map. This forms 3D-3D correspondences $C_{RGB-D}=\left\{\left(e_{i},y_{i}\right)\right\}$. We employ the Kabsch algorithm [50] to solve for the rigid transformation. In the sampling step, minimal subsets of 3 correspondences are required. The refinement step minimizes the direct Euclidean distance in 3D space:

(6) $$r_{RGB-D}\left(y_{i},h\right)={∥e_{i}-h^{-1}y_{i}∥}_{2}$$

### 4.4. Training Strategy and Loss Functions

To ensure stable convergence and high precision, we design a specific training strategy and loss function formulation, as summarized in Algorithm 2. We adopt a two-stage optimization scheme. In the first stage, the SCR network is initialized for 100 epochs by minimizing $L_{init}$, which provides stable supervision for scene coordinate regression. In the second stage, the DSAC* module is activated and the entire pipeline is optimized end-to-end for one epoch using the pose loss $L_{pose}$ together with the robustly scaled objective $L_{final}$ (with τ=100), thereby enabling gradients to propagate through the differentiable RANSAC procedure back to the SCR network.
**Algorithm 2** DOU-Pose training (warm-up initialization + end-to-end DSAC*)**Require:** Training samples $\left(I,K,h_{gt}\right)$, (optional) depth map; SCR net f(·;w); Adam; hypotheses *N*; soft inlier threshold; robust threshold τ; (RGB warm-up validity constraints).
**Ensure:** Trained w (DO-Conv foldable via Equation (3)).
  1:**Stage A (warm-up): minimize $L_{init}$**  2:**for** each warm-up iteration **do**  3:   $I\leftarrow G_{\mathrm{RAY}}(I)$; Y←f(I;w)  4:   **if** RGB-D **then**  5:       Compute GT scene coords $y_{i}^{*}$ from depth and $h_{gt}$.  6:     $L_{init}\leftarrow \frac{1}{\left|Y\right|}\sum_{i}{∥y_{i}^{*}-y_{i}∥}_{2}$                                                                                                                                                                                                                                                                                   (Equation (7))  7:   **else**  8:       Guided targets $y_{i}^{*}=h_{gt}e_{i}$                                                                                                                                                                                                                                                                             (Equation (1))  9:       Keep valid pixels by depth range and reprojection-error constraints.10:       $L_{init}\leftarrow$ mean reprojection error to $y_{i}^{*}$ (valid pixels).11:   **end if**12:     Adam update: $w\leftarrow A_{\mathrm{DAM}}(\nabla_{w}L_{init})$.13:**end for**14:Stage B (end-to-end): minimize $L_{final}$ through DSAC*15:**for** each end-to-end iteration **do**16:   $I\leftarrow G_{\mathrm{RAY}}(I)$; Y←f(I;w)17:   $h_{est}\leftarrow \mathrm{DSAC}*\left(Y,K,\mathrm{depth}?;N,\mathrm{thr}\right)$18:   $L_{pose}\leftarrow {∥t_{est}-t_{gt}∥}_{2}+\gamma \;∠\left(R_{est},R_{gt}\right)$                                                                                                                                                                                                                                                                    (Equation (8))19:   $L_{final}\leftarrow \left\{\begin{array}{cc} L_{pose} & L_{pose}<\tau \\ \sqrt{\tau \;L_{pose}} & \mathrm{otherwise} \end{array}\right.$                                                                                                                                                                                                                                                                           (Equation (9))20:   Adam update: $w\leftarrow A_{\mathrm{DAM}}(\nabla_{w}L_{final})$ (gradients through DSAC*).21:**end for**22:**Inference prep:** fold DO-Conv kernels using Equation (3).


#### 4.4.1. Network Initialization Strategy

Directly training the network with the pose loss from scratch can be unstable due to the randomness of RANSAC. Therefore, we implement a “warm-up” phase to initialize the scene coordinate regression network.

- **For RGB Inputs**: We use a “guided target” strategy. We define a pseudo-ground-truth scene coordinate $y_{i}^{*}=h_{gt}e_{i}$ using the ground truth pose $h_{gt}$ and a virtual camera coordinate $e_{i}$. To prevent the network from learning invalid correspondences, we apply strict validity constraints: a pixel is valid only if its virtual depth is between 1 m and 1000 m, and its initial reprojection error is less than 1000px. The initialization loss minimizes the reprojection error with respect to these valid targets.
- **For RGB-D Inputs**: The initialization is straightforward. We minimize the Euclidean distance between the predicted coordinate $y_{i}$ and the ground truth scene coordinate $y_{i}^{*}$ derived from the measured depth:
  (7) $$L_{init}=\frac{1}{\left|Y\right|}\sum_{y_{i}\in Y}{∥y_{i}^{*}-y_{i}∥}_{2}$$

#### 4.4.2. Pose Loss Function

After initialization, the network is trained end-to-end to minimize the pose error. The loss function $L_{pose}$ is a weighted combination of translation and rotation errors

(8) $$L_{pose}\left(h_{est},h_{gt}\right)={∥t_{est}-t_{gt}∥}_{2}+\gamma ∠\left(R_{est},R_{gt}\right)$$

where γ is a weighting factor balancing the two terms. We set γ=0.01, equivalent to the translation and rotation weights of $\lambda_{t}=100$ and $\lambda_{r}=1$, respectively. To improve robustness against outliers during training (e.g., when RANSAC fails completely), we apply robust scaling to the gradients. Specifically, if the loss value exceeds a threshold τ=100, we dampen the loss using a square-root formulation:

(9) $$L_{final}=\left\{\begin{array}{cc} L_{pose} & \mathrm{if}\;L_{pose}<\tau \\ \sqrt{\tau \cdot L_{pose}} & \mathrm{otherwise} \end{array}\right.$$

This mechanism prevents extreme gradients from destabilizing the network weights, effectively acting as a soft truncation for large errors.

## 5. Experiments

In this section, we evaluate DOU-Pose on standard indoor benchmarks and the self-collected Campus-AV dataset for real-world intelligent transportation scenarios.

### 5.1. Experimental Setup

#### 5.1.1. Datasets Description

**Figure 2:**
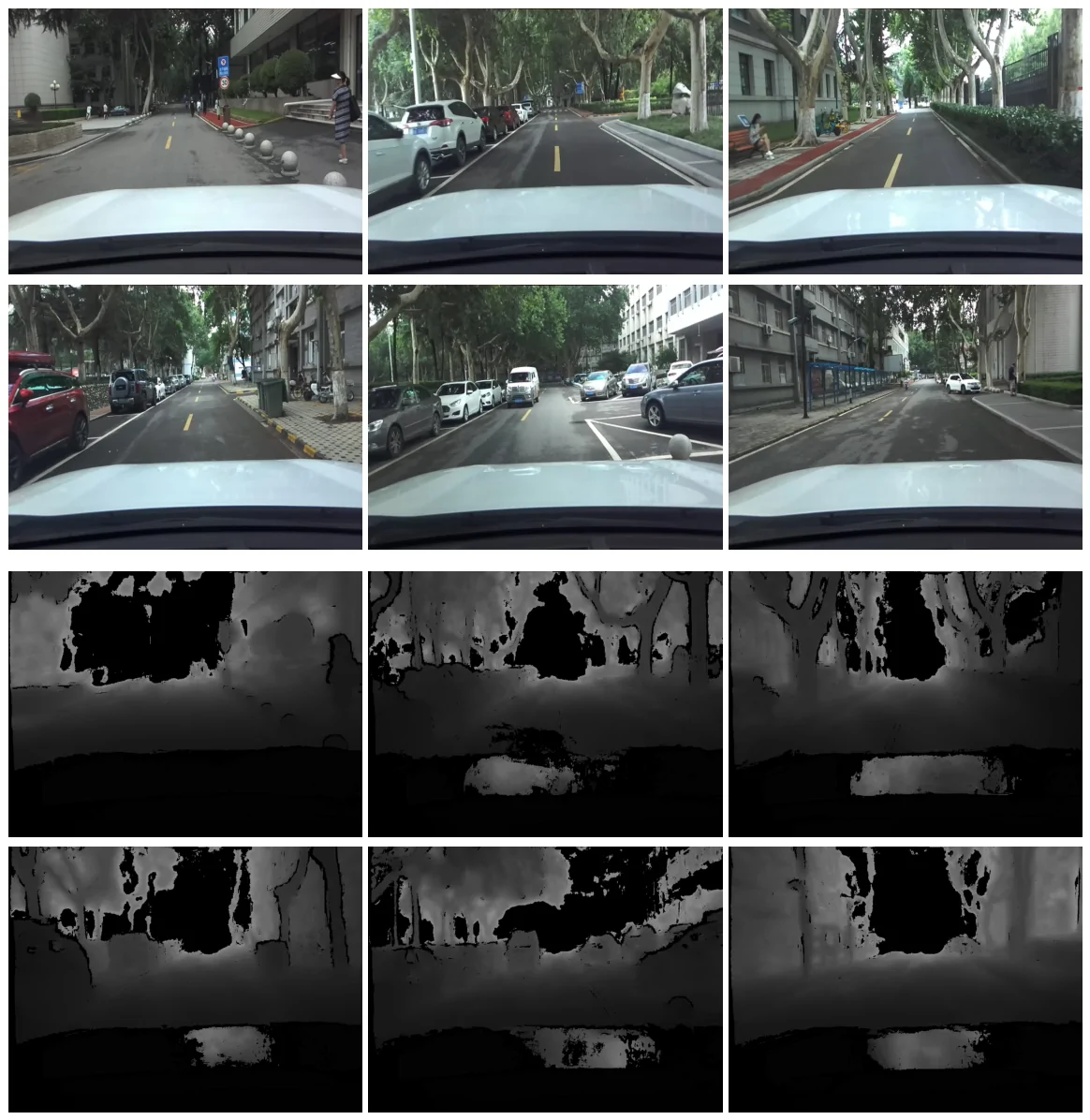
Sample RGB images and synchronized depth maps from the self-collected real-world Campus-AV dataset. The dataset was captured in a university campus environment using a connected autonomous vehicle platform, covering challenging road scenes with tree-lined roads, building facades, complex lighting, and structural ambiguities.

We evaluate the method on three datasets, ranging from standard indoor benchmarks to the outdoor Campus-AV driving dataset:

- **7-Scenes Dataset** [37]: A widely used indoor RGB-D benchmark recorded with a handheld Kinect [51] camera. It consists of seven scenes (e.g., *Chess*, *Stairs*, *Heads*) characterized by motion blur, repetitive geometric structures, and textureless surfaces.
- **12-Scenes Dataset** [52]: A moderately sized indoor dataset captured using Structure.io sensors. It features high-quality synchronized RGB-D frames and precise ground truth poses, covering varied domestic and office environments.
- **Campus-AV Dataset**: To bridge the gap between indoor benchmarks and practical ITS applications, we constructed an extensive real-world vehicular dataset, as illustrated by the RGB images and synchronized depth maps in Figure 2. Data collection was performed using a connected autonomous vehicle platform equipped with a ZED 2i stereo camera mounted behind the front rearview mirror. We configured the camera to a resolution of 1280×720 at 30 FPS, utilizing its wide 110° horizontal field of view to effectively capture road features. The vehicle traversed the campus, covering a trajectory of four complete loops through diverse environments, including tree-lined roads and building facades. Frame-level reference poses were obtained from the ZED SDK using zed.get_position() and stored as 4-by-4 camera-to-world transformations. The final dataset contains 50,000 synchronized frames and uses a loop-based partition. Loops 1–3, comprising 12,284, 12,671, and 12,545 frames, respectively, form the 37,500-frame training set, while Loop 4 provides a held-out test set of 12,500 frames. All four loops were recorded in one continuous localization session without resetting tracking and share the same local world coordinate system. The test loop contains no frames from the training loops, thereby avoiding frame overlap between the two sets. The corresponding depth maps allow the framework to operate in RGB-D mode by establishing 3D-3D correspondences through the back-projection of 2D pixels into camera space.

**Campus-AV pose reference and independent validation.** The saved transformation maps points from the left-camera coordinate system to the shared local world frame, with translation expressed in meters and the camera axes defined as x right, y down, and z forward. RGB images, depth maps, and ZED poses were recorded in the same SVO2 file and associated by frame timestamps at 30 Hz. Camera intrinsics were obtained using the ZED calibration tool, while the rotation and lever-arm translation of the camera-to-MTi extrinsic were calibrated jointly before trajectory validation. The independent MTi-680G RTK-GNSS/INS trajectory was recorded at 100 Hz. Synchronization used an affine clock model based on common host timestamps and was refined once on a predefined Loop-1 calibration segment using motion correlation; MTi translations were linearly interpolated and orientations were interpolated by quaternion SLERP. The maximum nearest timestamp difference was 9.70 ms, and the maximum interpolation interval was 19.40 ms. A single fixed unit-scale SE(3) rigid transform from the MTi ENU frame to the ZED world frame was estimated on the same Loop-1 calibration segment and applied to all four loops. Across all loops, independent validation yielded an absolute trajectory error RMSE of 0.66 m, a median absolute trajectory error of 0.48 m, and relative pose errors of 0.13 m per 1 s in translation and 0.68 degrees per 1 s in rotation, with 98.29% valid coverage. On the held-out Loop-4, the corresponding values were 0.77 m, 0.58 m, 0.16 m per 1 s, 0.79 degrees per 1 s, and 98.06%. The ZED poses serve as the training and test reference labels, whereas the MTi-680G trajectory is reserved for independent validation.

#### 5.1.2. Implementation Details

All experiments were conducted on a workstation equipped with an Intel Core i5-10400F CPU and an NVIDIA GeForce RTX 3070 GPU using Python 3.7, PyTorch 1.10.2, and CUDA 11.3. RGB inputs were converted to grayscale, resized to a height of 480 pixels while preserving the aspect ratio, and normalized after ToTensor using a mean of 0.4 and a standard deviation of 0.25; no random crop was applied. Campus-AV images were therefore approximately 853-by-480 pixels. During training, we employed the Adam optimizer [53] with a constant learning rate of $10^{-3}$, coefficients of (0.9, 0.999), epsilon of 1 × 10^−8^, and zero weight decay, and a batch size of 1. Training used rotation, scale, brightness, and contrast augmentation, with random seeds 0, 1, and 2. The network was trained from scratch without external pretrained weights. For the differentiable RANSAC configuration, the number of hypothesis samples was set to 64, and the soft inlier threshold was set to 10 pixels. Regarding the robust loss function, the soft square-root threshold was set to τ=100, while the hard truncation threshold was set to 1000 pixels to prevent gradient explosion from extreme outliers.

#### 5.1.3. Evaluation Metrics

To measure the localization performance, we report the mean errors for translation and rotation:

(10) $$E_{trans}=\left|\right|t_{est}-t_{gt}{\left|\right|}_{2},\;E_{rot}=∠\left(R_{est},R_{gt}\right)$$

Additionally, strictly following the established evaluation protocol for the 7-Scenes and 12-Scenes benchmarks [2,3,37,52], we report the accuracy, defined as the percentage of test frames where the translation error is below 5cm and the rotation error is below 5°.

### 5.2. Performance on Public Benchmarks

We evaluate DOU-Pose on the 7-Scenes and 12-Scenes indoor benchmarks. The comparison includes PoseNet, DSAC++, DSAC*, and the recent SCR baseline ACE [1,2,3,42].

#### 5.2.1. Translation and Rotation Errors

We assess the metric accuracy of the estimated poses by reporting the median translation and rotation errors over the test frames of each scene; the Avg. row averages the seven scene-wise medians.

Table 1 reports the scene-wise median errors on 7-Scenes. Averaging the seven scene-wise medians, DOU-Pose obtains translation and rotation errors of 2.81 cm and 0.90°, compared with 2.84 cm and 0.96° for DSAC*. The largest scene-level differences occur on *Heads*, where the errors decrease from 1.2 cm and 0.7° to 1.1 cm and 0.6°, and on *Stairs*, where the rotation error decreases from 1.3° to 1.2°.

#### 5.2.2. Localization Accuracy

To evaluate the robustness of the system, we report the localization accuracy, defined as the percentage of frames with a translation error below 5 cm and a rotation error below 5° [2,3,37].

Table 2 shows an average 7-Scenes accuracy of 81.48% for DOU-Pose and 78.23% for DSAC*, a difference of 3.25 percentage points. The largest scene-level differences are observed on *Heads* (99.4% versus 92.4%) and *Stairs* (64.2% versus 56.3%).

**Figure 3:**
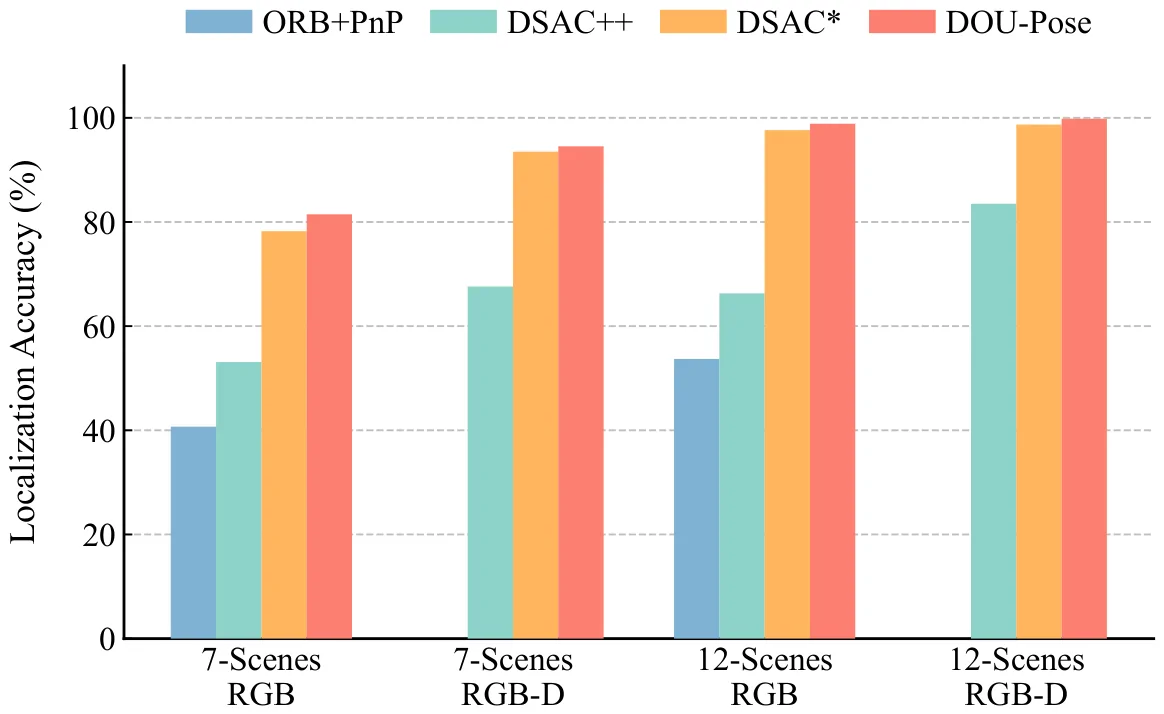
Localization accuracy of ORB+PnP, DSAC++, DSAC*, and DOU-Pose on 7-Scenes and 12-Scenes using RGB and RGB-D inputs. Accuracy denotes the percentage of test frames satisfying the 5 cm/5-degree criterion.

The aggregate comparison in Table 3 places DOU-Pose close to both DSAC* and ACE. On 7-Scenes, its averages of the scene-wise median translation and rotation errors are 2.81 cm and 0.90°, respectively, and its localization accuracy is 81.48%. The corresponding 12-Scenes values are 1.03 cm, 0.44°, and 99.50%. These margins support competitive performance on the public indoor benchmarks. With RGB-D input, DOU-Pose obtains localization accuracies of 94.53% on 7-Scenes and 99.83% on 12-Scenes. Figure 3 visualizes these results together with the corresponding ORB+PnP and DSAC++ baselines.

### 5.3. Performance on Real-World Campus-AV Dataset

To validate the generalization capability and robustness of the proposed DOU-Pose in real-world autonomous driving scenarios, we conducted extensive evaluations on the self-collected Campus-AV dataset. Unlike the indoor benchmarks, this dataset features complex outdoor lighting, large-scale motions, and diverse structural ambiguities.

#### 5.3.1. Evaluation Protocol and Overall Results

The strict and relaxed success thresholds were fixed at 2 m/5° and 5 m/10°, respectively, before inspecting the rerun results. DSAC*, DOU-Pose, and ACE were each trained with seeds 0, 1, and 2, and every run was evaluated on the complete 12,500-frame Loop-4 test sequence. The median and P90 errors summarize the central and upper-tail behavior of the complete test sequence, while the strict and relaxed success rates report the percentages of frames satisfying the two fixed translation–rotation thresholds. Table 4 summarizes the overall Campus-AV results over three training seeds, while Table 5 further reports marginal cumulative rates at fixed translation-only and rotation-only thresholds.

At translation thresholds of 1, 2, and 5 m, the cumulative rates increase from 3.9%, 15.1%, and 59.8% for DSAC* to 6.2%, 22.8%, and 69.1% for DOU-Pose. Across rotation thresholds of 2, 5, and 10 degrees, the corresponding rates increase from 4.7%, 32.9%, and 76.8% to 6.5%, 39.3%, and 81.4%.

DOU-Pose reduces the mean translation error from 5.410 m to 4.803 m and the mean rotation error from 7.597° to 7.260°, corresponding to relative reductions of 11.2% and 4.4%. The strict success rate increases from 10.8% to 16.8%, while the relaxed success rate increases from 53.3% to 62.8%. Compared with ACE, DOU-Pose reduces the mean translation error from 5.100 m to 4.803 m and the mean rotation error from 7.430° to 7.260°, while increasing the relaxed success rate from 58.1% to 62.8%. Using the three DSAC* seeds and three DOU-Pose seeds as the unit of analysis, two-sided Welch’s *t*-tests give p=0.00565 for the seed-level mean translation errors and p=0.00835 for the mean rotation errors.

#### 5.3.2. Per-Frame Error Curves in a Challenging Sequence

**Figure 4:**
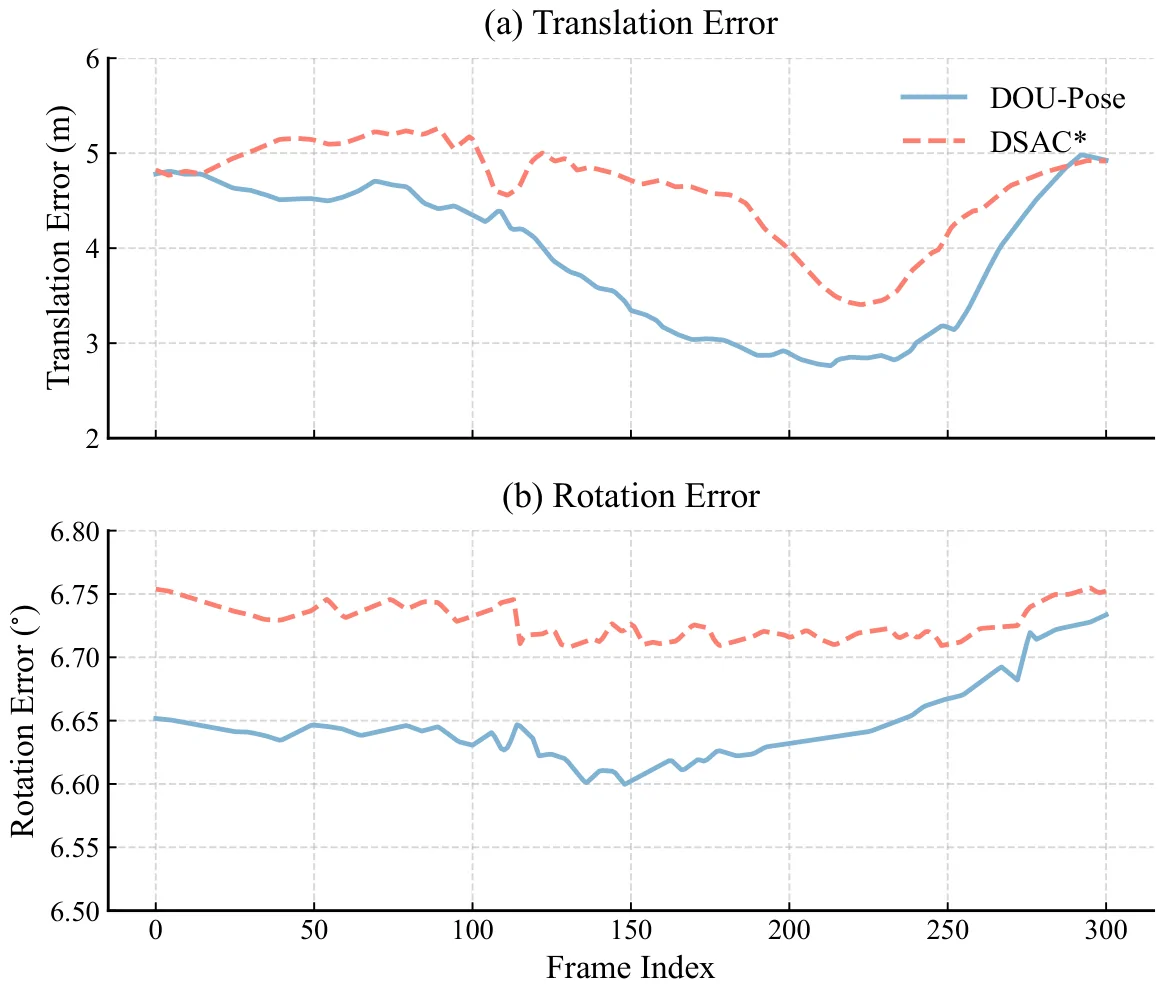
Per-frame translation (**a**) and rotation (**b**) errors of DSAC* and DOU-Pose over Loop-4 frames 003450–003749. Frames 003450–003619 contain repetitive structures, and frames 003620–003749 contain a low-texture region. DOU-Pose follows a lower rotation-error trajectory throughout the segment and a lower translation-error trajectory over most frames, with the clearest translation gap around the transition between the two portions.

Figure 4 presents the frame-wise translation and rotation error traces of DSAC* and DOU-Pose on the contiguous Loop-4 segment from frame 003450 to frame 003749. The segment was fixed after the loop split and before per-frame evaluation using its predefined road location and scene content. Frames 003450–003619 cover repeated building facades, regularly arranged windows, and continuous metal railings, while frames 003620–003749 cover a low-texture light-colored wall. The curves characterize localization behavior across these consecutive repetitive-structure and low-texture portions.

### 5.4. Ablation Studies

The proposed method incorporates Depthwise Over-parameterized Convolution (DO-Conv) and transposed convolutions into the baseline DSAC* framework. To evaluate the contribution of each component, we conduct an ablation study on the 7-Scenes dataset using the accuracy threshold of <5 cm and 5°. We compare four configurations: the baseline model $M_{base}$, a DO-Conv variant $M_{do}$, a transposed-convolution variant $M_{trans}$, and the full model $M_{full}$.

As shown in Table 6, both DO-Conv and transposed convolutions improve localization accuracy over the baseline, and the full model achieves the best average result of 81.5%. Introducing DO-Conv increases the average accuracy from 78.2% to 81.0%, with clear gains in challenging scenes such as *Heads* (92.4%→98.3%) and *Stairs* (56.3%→63.0%). The transposed-convolution variant also improves the average accuracy to 80.5%, and further combining it with DO-Conv increases the accuracy from 81.0% to 81.5%, indicating that the two components are complementary. Overall, DO-Conv strengthens feature representation, while transposed convolutions improve spatial-detail recovery, and their combination achieves the best average localization accuracy among the four evaluated configurations.

The Campus-AV ablation in Table 7 shows consistent contributions from both components. Relative to $M_{base}$, adding DO-Conv increases the strict and relaxed success rates from 10.8% and 53.4% to 14.6% and 59.3%, while the transposed-convolution variant reaches 12.9% and 56.7%. Combining both components yields the lowest median and P90 errors, together with the highest strict and relaxed success rates of 16.7% and 62.8%. These results extend the component analysis to the target outdoor setting.

### 5.5. Model Complexity and Efficiency

Table 8 quantifies the computation–robustness trade-off. Folding the auxiliary DO-Conv parameterization reduces the DOU-Pose graph from 11.42 M to 11.09 M parameters and the mean latency from 58.96 ms to 57.21 ms. The folded model operates at 17.48 FPS, compared with 41.37 FPS for DSAC*, while providing the Campus-AV improvements reported in Table 4.

## 6. Conclusions

In this paper, we proposed DOU-Pose, a visual pose estimation framework that integrates Depthwise Over-parameterized Convolutions (DO-Conv) and a U-shaped architecture to improve scene-coordinate prediction under perceptual aliasing and feature sparsity in autonomous driving. Experiments show competitive results against strong baselines on public indoor benchmarks and improved Campus-AV metrics relative to DSAC*, including repetitive and low-texture outdoor sequences. Future work will focus on further enhancing system robustness by expanding the dataset to include diverse weather conditions and incorporating dynamic object filtering modules to ensure reliable localization in complex, dynamic environments.

## Figures and Tables

**Table 1:** Comparison of median translation (cm) and rotation (°) errors on the 7-Scenes Dataset.

Scene	PoseNet		DSAC++		DSAC*		DOU-Pose	
	Trans.	Rot.	Trans.	Rot.	Trans.	Rot.	Trans.	Rot.
Chess	30	4.1	2.0	**0.5**	**1.9**	0.6	**1.9**	0.6
Fire	49	7.3	2.0	0.9	**1.9**	0.9	**1.9**	**0.8**
Heads	28	6.0	**1.0**	0.8	1.2	0.7	1.1	**0.6**
Office	47	3.8	3.0	**0.7**	**2.6**	0.8	**2.6**	**0.7**
Pumpkin	46	4.2	**4.0**	**1.1**	4.1	**1.1**	4.1	**1.1**
RedKitchen	62	4.3	4.0	**1.1**	**3.9**	1.3	**3.9**	1.3
Stairs	47	6.9	9.0	2.6	4.3	1.3	**4.2**	**1.2**
**Avg.**	44.1	5.2	3.6	1.1	2.8	1.0	**2.8**	**0.9**

**Table 2:** Localization accuracy (<5 cm, 5°) on 7-Scenes Dataset.

Scene	DSAC* (%)	DOU-Pose (%)
Chess	**97.1**	97.0
Fire	89.6	**92.7**
Heads	92.4	**99.4**
Office	86.6	**89.3**
Pumpkin	59.0	**61.2**
RedKitchen	**66.6**	**66.6**
Stairs	56.3	**64.2**
**Average**	78.23	**81.48**

**Table 3:** Aggregate RGB comparison on public benchmarks. Translation and rotation entries are averages of scene-wise median errors, while accuracy is the percentage of test frames below 5 cm and 5°. ACE entries are published mean ± standard deviation across runs [42]. Lower values are better for metrics marked with ↓, while higher values are better for metrics marked with ↑.

Dataset	Method	Avg. Median T (cm) ↓	Avg. Median R (°) ↓	Acc. (%) ↑
**7-Scenes**	DSAC*	2.84	0.96	78.23
	ACE	2.93±0.03	0.99±0.02	80.70±0.36
	**DOU-Pose**	**2.81**	**0.90**	**81.48**
**12-Scenes**	DSAC*	1.10	0.50	98.64
	ACE	1.05±0.03	0.49±0.04	98.87±0.10
	**DOU-Pose**	**1.03**	**0.44**	**99.50**

**Table 4:** Campus-AV results over three training seeds. Available entries are reported as mean ± sample standard deviation across seeds; dashes mark ACE metrics outside the reported aggregate.

Method	Mean T (m) ↓	Median T	P90 T	Mean R (°) ↓	Median R	P90 R	SR@2m/ 5° (%) ↑	SR@5m/ 10° (%) ↑
DSAC*	5.410±0.131	4.677±0.156	9.130±0.236	7.597±0.090	6.863±0.150	12.457±0.296	10.8±0.9	53.3±1.6
**DOU-Pose**	4.803±0.142	4.053±0.078	8.000±0.207	7.260±0.075	6.513±0.064	11.810±0.213	16.8±1.1	62.8±1.3
ACE	5.100±0.040	–	–	7.430±0.080	–	–	–	58.1±1.3

**Table 5:** Campus-AV marginal cumulative error statistics reported as mean ± sample standard deviation across three seeds under the complete Loop-4 evaluation protocol. Translation and rotation thresholds are evaluated independently.

Error Type	Method	Median ↓	P90 ↓	Threshold 1 ↑	Threshold 2 ↑	Threshold 3 ↑
**Translation (m)**	DSAC*	4.677±0.156	9.130±0.236	3.9±0.3	15.1±0.6	59.8±0.7
	**DOU-Pose**	4.053±0.078	8.000±0.207	6.2±0.3	22.8±0.8	69.1±0.8
**Rotation (°)**	DSAC*	6.863±0.150	12.457±0.296	4.7±0.2	32.9±0.6	76.8±0.7
	**DOU-Pose**	6.513±0.064	11.810±0.213	6.5±0.4	39.3±0.7	81.4±0.7

*Note:* For translation, Thresholds 1–3 denote T≤1m, T≤2m, and T≤5m, respectively. For rotation, they denote R≤2°, R≤5°, and R≤10°. Threshold results are reported in percent.

**Table 6:** Ablation study of different modules on the 7-Scenes dataset (accuracy <5 cm, 5°).

Scene	$M_{base}$	$M_{do}$	$M_{trans}$	$M_{full}$
Chess	**97.1**	**97.1**	96.8	97.0
Fire	89.6	92.1	91.9	**92.7**
Heads	92.4	98.3	98.8	**99.4**
Office	86.6	88.9	88.1	**89.3**
Pumpkin	59.0	60.8	60.1	**61.2**
RedKitchen	**66.6**	66.5	66.5	**66.6**
Stairs	56.3	63.0	61.2	**64.2**
**Avg.**	78.2	81.0	80.5	**81.5**

**Table 7:** Component ablation on Campus-AV using seed 0, the complete Loop-4 test sequence, and the fixed success thresholds.

Config.	DO-Conv	Transposed Conv.	Median T (m) ↓	P90 T (m) ↓	Median R (°) ↓	P90 R (°) ↓	SR@2m/ 5° (%) ↑	SR@5m/ 10° (%) ↑
$M_{base}$	No	No	4.73	9.12	6.85	12.46	10.8	53.4
$M_{do}$	Yes	No	4.31	8.43	6.68	12.04	14.6	59.3
$M_{trans}$	No	Yes	4.49	8.76	6.71	11.97	12.9	56.7
$M_{full}$	Yes	Yes	**4.05**	**8.01**	**6.52**	**11.84**	**16.7**	**62.8**

**Table 8:** Model complexity and inference efficiency on an RTX 3070 at 853-by-480 input resolution, batch size 1, over 500 timing runs.

Method/Graph	Params (M)	GFLOPs	Mean Latency (ms)	P90 Latency (ms)	FPS	Peak VRAM (GB)
DSAC*	6.85	94.91	24.18	27.06	41.37	1.18
DOU-Pose training graph	11.42	331.31	58.96	64.73	16.96	1.69
DOU-Pose folded inference	11.09	331.22	57.21	62.53	17.48	1.63

## Data Availability

The Campus-AV dataset generated and analyzed during the current study is available from the corresponding author upon reasonable request. Public benchmark datasets used in this study are available from their respective official sources.

## Abbreviations

The following abbreviations are used in this manuscript:

DO-Conv	Depthwise Over-parameterized Convolution
DOU-Pose	Depthwise Over-parameterized U-shaped Pose estimation
DSAC	Differentiable Sample Consensus
GNSS	Global Navigation Satellite System
IMU	Inertial Measurement Unit
ITS	Intelligent Transportation Systems

PnP	Perspective-n-Point
RGB-D	Red–Green–Blue-Depth
SCR	Scene Coordinate Regression
SfM	Structure-from-Motion
VO	Visual Odometry
